# 5-Cyclo­hexyl-2-methyl-3-(3-methyl­phenyl­sulfon­yl)-1-benzo­furan

**DOI:** 10.1107/S1600536814006448

**Published:** 2014-03-29

**Authors:** Hong Dae Choi, Pil Ja Seo, Uk Lee

**Affiliations:** a Department of Chemistry, Dongeui University, San 24 Kaya-dong, Busanjin-gu, Busan 614-714, Republic of Korea; b Department of Chemistry, Pukyong National University, 599-1 Daeyeon 3-dong, Nam-gu, Busan 608-737, Republic of Korea

## Abstract

In the title compound, C_22_H_24_O_3_S, the cyclo­hexyl ring adopts a chair conformation. The dihedral angle between the mean plane [r.m.s. deviation = 0.010 (1) Å] of the benzo­furan ring system and the benzene ring is 81.78 (4)°. In the crystal, mol­ecules are linked *via* pairs of C—H⋯π inter­actions into inversion dimers. These dimers are further linked by C—H⋯π inter­actions into supra­molecular chains running along the *b-*axis direction. In addition, C—H⋯O hydrogen bonds are observed between inversion-related dimers.

## Related literature   

For background information and the crystal structures of related compounds, see: Choi *et al.* (2011 ▶, 2012*a* ▶,*b* ▶).
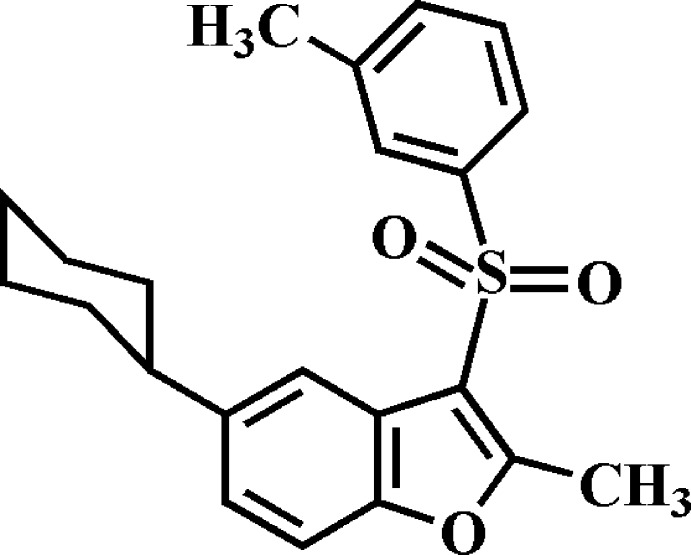


## Experimental   

### 

#### Crystal data  

C_22_H_24_O_3_S
*M* *_r_* = 368.47
Triclinic, 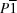

*a* = 8.9729 (1) Å
*b* = 10.3462 (1) Å
*c* = 11.0978 (2) Å
α = 91.027 (1)°
β = 112.142 (1)°
γ = 96.920 (1)°
*V* = 945.09 (2) Å^3^
*Z* = 2
Mo *K*α radiation
μ = 0.19 mm^−1^
*T* = 173 K
0.45 × 0.24 × 0.12 mm

#### Data collection  

Bruker SMART APEXII CCD diffractometer
Absorption correction: multi-scan (*SADABS*; Bruker, 2009 ▶) *T*_min_ = 0.704, *T*_max_ = 0.746
17505 measured reflections
4676 independent reflections
3862 reflections with *I* > 2σ(*I*)
*R*_int_ = 0.027

#### Refinement  

*R*[*F* ^2^ > 2σ(*F* ^2^)] = 0.042
*wR*(*F* ^2^) = 0.117
*S* = 1.03
4676 reflections
237 parameters
H-atom parameters constrained
Δρ_max_ = 0.27 e Å^−3^
Δρ_min_ = −0.36 e Å^−3^

### 

Data collection: *APEX2* (Bruker, 2009 ▶); cell refinement: *SAINT* (Bruker, 2009 ▶); data reduction: *SAINT*; program(s) used to solve structure: *SHELXS97* (Sheldrick, 2008 ▶); program(s) used to refine structure: *SHELXL97* (Sheldrick, 2008 ▶); molecular graphics: *ORTEP-3 for Windows* (Farrugia, 2012 ▶) and *DIAMOND* (Brandenburg, 1998 ▶); software used to prepare material for publication: *SHELXL97*.

## Supplementary Material

Crystal structure: contains datablock(s) I. DOI: 10.1107/S1600536814006448/kj2238sup1.cif

Structure factors: contains datablock(s) I. DOI: 10.1107/S1600536814006448/kj2238Isup2.hkl

Supporting information file. DOI: 10.1107/S1600536814006448/kj2238Isup3.cml

CCDC reference: 993236

Additional supporting information:  crystallographic information; 3D view; checkCIF report

## Figures and Tables

**Table 1:** Hydrogen-bond geometry (Å, °) *Cg*1 and *Cg*2 are the centroids of the C2–C7 benzene ring and the C1/C2/C7/O1/C8 furan ring, respectively

*D*—H⋯*A*	*D*—H	H⋯*A*	*D*⋯*A*	*D*—H⋯*A*
C17—H17⋯O2^i^	0.95	2.55	3.415 (2)	151
C13—H13*B*⋯*Cg*1^ii^	0.99	2.83	3.670 (2)	143
C19—H19⋯*Cg*2^iii^	0.95	2.85	3.705 (2)	150

Symmetry codes: (i) 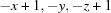; (ii) 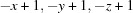; (iii) 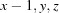.

**Fig. 1.**
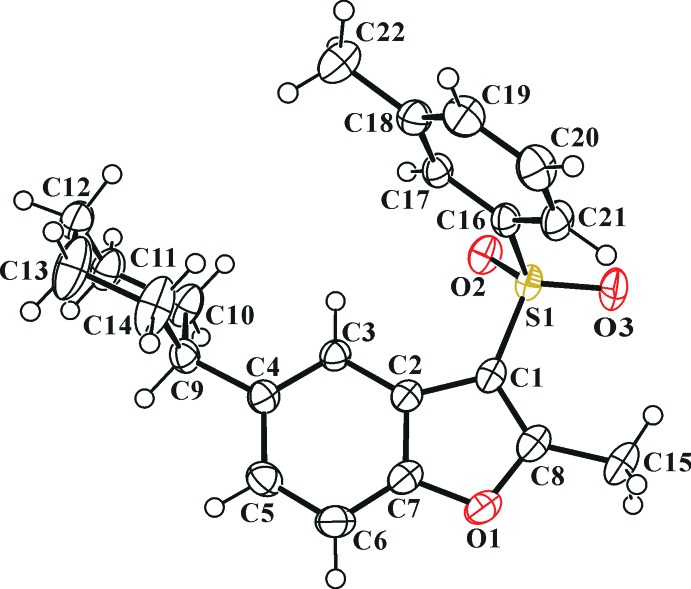
The molecular structure of the title compound with the atom numbering scheme. Displacement ellipsoids are drawn at the 50% probability level. H atoms are presented as small spheres of arbitrary radius.

**Fig. 2.**
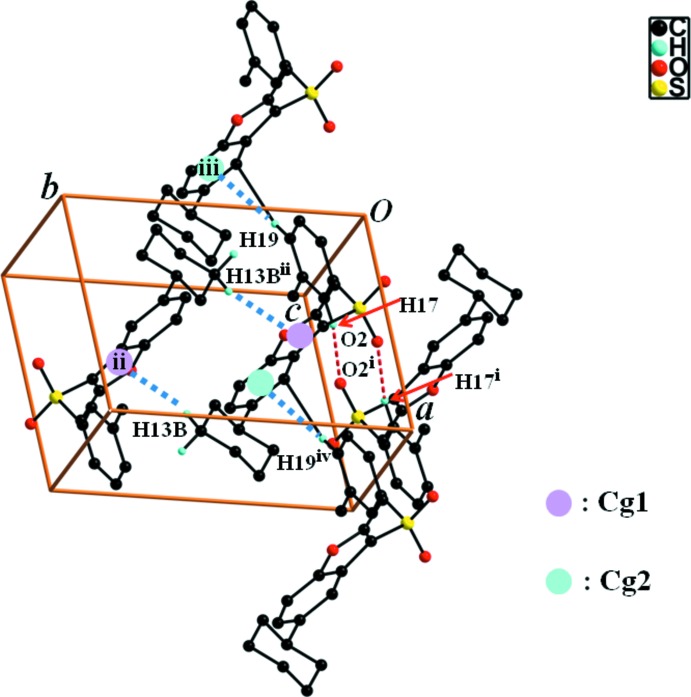
A view of the C—H···O and C—H···π hydrogen bonds (dotted lines) in the crystal structure of the title compound. H atoms non-participating in hydrogen-bonding were omitted for clarity. [Symmetry codes : (i) - x + 1, - y, - z + 1; (ii) - x + 1, - y + 1, - z + 1; (iii) x - 1, y, z; (iv) x + 1, y, z.]

## supplementary crystallographic information

### 1. Comment

As a part of our continuing study of 5-cyclohexyl-2-methyl-1-benzofuran derivatives containing 4-fluorophenylsulfonyl (Choi *et al.*, 2011), 4-bromophenylsulfonyl (Choi *et al.*, 2012*a*) and 4-methylphenylsulfonyl (Choi *et al.*, 2012*b*) substituents in 3-position, we report here the crystal structure of the title compound.

In the title molecule (Fig. 1), The cyclohexyl ring has a chair conformation. The benzofuran ring system is essentially planar, with a mean deviation of 0.010 (1) Å from the least-squares plane defined by the nine constituent atoms. The 3-methylphenyl ring is essentially planar, with a mean deviation of 0.005 (1) Å from the least-squares plane defined by the six constituent atoms. The dihedral angle formed by the benzofuran ring system and the 3-methylphenyl ring is 81.78 (4)°. In the crystal structure (Fig. 2), molecules are connected by pairs of C—H···π interactions into inversion dimers (Table 1; Cg1 is the centroid of the C1/C2/C7/O1/C8 furan ring). These dimers are further linked by C—H···π interactions into supramolecular chains running along the *b*-axis (Table 1; Cg2 is the centroid of the C2–C7 benzene ring). In addition, there are C–H···O hydrogen bonds (Table 1), resulting in inversion-related dimers.

### 2. Experimental

3-Chloroperoxybenzoic acid (77%, 448 mg, 2.0 mmol) was added in small portions to a stirred solution of 5-cyclohexyl-2-methyl-3-(3-methylphenylsulfanyl)-1-benzofuran (302 mg, 0.9 mmol) in dichloromethane (40 mL) at 273 K. After being stirred at room temperature for 10h, the mixture was washed with saturated sodium bicarbonate solution and the organic layer was separated, dried over magnesium sulfate, filtered and concentrated at reduced pressure. The residue was purified by column chromatography (hexane–ethyl acetate, 4:1 *v*/*v*) to afford the title compound as a colorless solid [yield 73%, m.p. 428–429 K; *R*_f_ = 0.57 (hexane–ethyl acetate, 4:1 v/v)]. Single crystals suitable for X-ray diffraction were prepared by slow vaporation of a solution of the title compound in benzene at room temperature.

### 3. Refinement

All H atoms were positioned geometrically and refined using a riding model, with C—H = 0.95 Å for aryl, 1.00 Å for methine, 0.99 Å for methylene and 0.98 Å for methyl H atoms, respectively. *U*_iso_ (H) = 1.2*U*_eq_ (C) for aryl, methine and methylene, and 1.5*U*_eq_ (C) for methyl H atoms. The positions of methyl hydrogens were optimized rotationally.

### Crystal data

**Table:**

C_22_H_24_O_3_S	*Z* = 2
*M**_r_* = 368.47	*F*(000) = 392
Triclinic, *P*1	*D*_x_ = 1.295 Mg m^−^^3^
Hall symbol: -P 1	Melting point = 428–429 K
*a* = 8.9729 (1) Å	Mo *K*α radiation, λ = 0.71073 Å
*b* = 10.3462 (1) Å	Cell parameters from 6413 reflections
*c* = 11.0978 (2) Å	θ = 2.5–28.0°
α = 91.027 (1)°	µ = 0.19 mm^−^^1^
β = 112.142 (1)°	*T* = 173 K
γ = 96.920 (1)°	Block, colourless
*V* = 945.09 (2) Å^3^	0.45 × 0.24 × 0.12 mm

### Data collection

**Table:**

Bruker SMART APEXII CCD diffractometer	4676 independent reflections
Radiation source: rotating anode	3862 reflections with *I* > 2σ(*I*)
Graphite multilayer monochromator	*R*_int_ = 0.027
Detector resolution: 10.0 pixels mm^-1^	θ_max_ = 28.4°, θ_min_ = 2.0°
φ and ω scans	*h* = −11→11
Absorption correction: multi-scan (*SADABS*; Bruker, 2009)	*k* = −13→13
*T*_min_ = 0.704, *T*_max_ = 0.746	*l* = −14→14
17505 measured reflections	

### Refinement

**Table:**

Refinement on *F*^2^	Primary atom site location: structure-invariant direct methods
Least-squares matrix: full	Secondary atom site location: difference Fourier map
*R*[*F*^2^ > 2σ(*F*^2^)] = 0.042	Hydrogen site location: difference Fourier map
*wR*(*F*^2^) = 0.117	H-atom parameters constrained
*S* = 1.03	*w* = 1/[σ^2^(*F*_o_^2^) + (0.0576*P*)^2^ + 0.3454*P*] where *P* = (*F*_o_^2^ + 2*F*_c_^2^)/3
4676 reflections	(Δ/σ)_max_ < 0.001
237 parameters	Δρ_max_ = 0.27 e Å^−^^3^
0 restraints	Δρ_min_ = −0.36 e Å^−^^3^

### Special details

**Table:**

Geometry. All esds (except the esd in the dihedral angle between two l.s. planes) are estimated using the full covariance matrix. The cell esds are taken into account individually in the estimation of esds in distances, angles and torsion angles; correlations between esds in cell parameters are only used when they are defined by crystal symmetry. An approximate (isotropic) treatment of cell esds is used for estimating esds involving l.s. planes.
Refinement. Refinement of F^2^ against ALL reflections. The weighted R-factor wR and goodness of fit S are based on F^2^, conventional R-factors R are based on F, with F set to zero for negative F^2^. The threshold expression of F^2^ > 2sigma(F^2^) is used only for calculating R-factors(gt) etc. and is not relevant to the choice of reflections for refinement. R-factors based on F^2^ are statistically about twice as large as those based on F, and R- factors based on ALL data will be even larger.

### Fractional atomic coordinates and isotropic or equivalent isotropic displacement parameters (Å^2^)

**Table:**

	*x*	*y*	*z*	*U*_iso_*/*U*_eq_	
S1	0.35800 (4)	0.03292 (4)	0.20843 (3)	0.02902 (11)	
O1	0.55693 (14)	0.33589 (12)	0.08723 (11)	0.0373 (3)	
O2	0.46948 (14)	−0.02883 (11)	0.31363 (10)	0.0359 (3)	
O3	0.27359 (15)	−0.03917 (11)	0.08444 (11)	0.0405 (3)	
C1	0.46417 (18)	0.17522 (15)	0.18450 (14)	0.0290 (3)	
C2	0.57504 (17)	0.26991 (14)	0.28633 (14)	0.0275 (3)	
C3	0.63168 (17)	0.28333 (14)	0.42235 (14)	0.0279 (3)	
H3	0.5980	0.2181	0.4694	0.033*	
C4	0.73853 (18)	0.39425 (15)	0.48773 (15)	0.0299 (3)	
C5	0.78770 (19)	0.48851 (16)	0.41575 (16)	0.0369 (4)	
H5	0.8608	0.5636	0.4613	0.044*	
C6	0.7337 (2)	0.47625 (17)	0.28097 (17)	0.0389 (4)	
H6	0.7682	0.5404	0.2333	0.047*	
C7	0.62752 (19)	0.36615 (16)	0.22028 (15)	0.0327 (3)	
C8	0.45740 (19)	0.22001 (16)	0.06823 (15)	0.0338 (3)	
C9	0.79994 (18)	0.41730 (15)	0.63454 (14)	0.0310 (3)	
H9	0.8961	0.4868	0.6611	0.037*	
C10	0.8576 (2)	0.29841 (18)	0.70674 (16)	0.0426 (4)	
H10A	0.9431	0.2692	0.6810	0.051*	
H10B	0.7657	0.2266	0.6813	0.051*	
C15	0.3672 (2)	0.1726 (2)	−0.07082 (16)	0.0449 (4)	
H15A	0.3005	0.0889	−0.0758	0.067*	
H15B	0.4446	0.1613	−0.1119	0.067*	
H15C	0.2970	0.2365	−0.1163	0.067*	
C16	0.21417 (17)	0.08713 (14)	0.26325 (14)	0.0274 (3)	
C17	0.24711 (17)	0.09527 (14)	0.39576 (14)	0.0283 (3)	
H17	0.3450	0.0700	0.4555	0.034*	
C18	0.13676 (19)	0.14042 (15)	0.44124 (15)	0.0325 (3)	
C19	−0.0052 (2)	0.17699 (17)	0.35005 (17)	0.0391 (4)	
H19	−0.0811	0.2095	0.3795	0.047*	
C20	−0.0379 (2)	0.16714 (18)	0.21828 (17)	0.0410 (4)	
H20	−0.1362	0.1918	0.1584	0.049*	
C21	0.07095 (19)	0.12176 (16)	0.17273 (15)	0.0350 (3)	
H21	0.0488	0.1143	0.0820	0.042*	
C22	0.1720 (2)	0.1479 (2)	0.58506 (17)	0.0450 (4)	
H22A	0.2309	0.0759	0.6250	0.068*	
H22B	0.0696	0.1414	0.5986	0.068*	
H22C	0.2385	0.2312	0.6253	0.068*	
C11	0.9247 (2)	0.32679 (18)	0.85430 (16)	0.0436 (4)	
H11A	0.9557	0.2458	0.8976	0.052*	
H11B	1.0235	0.3921	0.8810	0.052*	
C12	0.8014 (2)	0.3777 (2)	0.89752 (17)	0.0467 (4)	
H12A	0.8507	0.4010	0.9926	0.056*	
H12B	0.7080	0.3085	0.8802	0.056*	
C13	0.7425 (3)	0.4959 (2)	0.82666 (19)	0.0627 (6)	
H13A	0.8335	0.5684	0.8525	0.075*	
H13B	0.6564	0.5237	0.8526	0.075*	
C14	0.6753 (3)	0.4684 (2)	0.67829 (18)	0.0586 (6)	
H14A	0.5764	0.4032	0.6511	0.070*	
H14B	0.6447	0.5497	0.6355	0.070*	

### Atomic displacement parameters (Å^2^)

**Table:**

	*U*^11^	*U*^22^	*U*^33^	*U*^12^	*U*^13^	*U*^23^
S1	0.0321 (2)	0.03056 (19)	0.02058 (18)	0.00720 (14)	0.00497 (14)	−0.00076 (13)
O1	0.0391 (6)	0.0488 (7)	0.0271 (6)	0.0072 (5)	0.0157 (5)	0.0078 (5)
O2	0.0390 (6)	0.0375 (6)	0.0295 (6)	0.0160 (5)	0.0080 (5)	0.0054 (5)
O3	0.0492 (7)	0.0387 (6)	0.0258 (6)	0.0042 (5)	0.0066 (5)	−0.0077 (5)
C1	0.0287 (7)	0.0357 (8)	0.0227 (7)	0.0086 (6)	0.0087 (5)	0.0006 (6)
C2	0.0241 (7)	0.0332 (7)	0.0260 (7)	0.0070 (5)	0.0095 (5)	0.0027 (6)
C3	0.0259 (7)	0.0322 (7)	0.0246 (7)	0.0044 (5)	0.0083 (5)	0.0033 (5)
C4	0.0254 (7)	0.0331 (8)	0.0289 (7)	0.0050 (6)	0.0074 (6)	0.0027 (6)
C5	0.0314 (8)	0.0361 (8)	0.0381 (9)	−0.0001 (6)	0.0089 (7)	0.0053 (7)
C6	0.0347 (8)	0.0439 (9)	0.0384 (9)	0.0020 (7)	0.0149 (7)	0.0118 (7)
C7	0.0303 (7)	0.0429 (9)	0.0272 (7)	0.0091 (6)	0.0122 (6)	0.0061 (6)
C8	0.0337 (8)	0.0441 (9)	0.0258 (7)	0.0117 (6)	0.0120 (6)	0.0033 (6)
C9	0.0284 (7)	0.0308 (7)	0.0273 (7)	0.0016 (6)	0.0041 (6)	0.0009 (6)
C10	0.0561 (11)	0.0430 (9)	0.0298 (8)	0.0213 (8)	0.0131 (7)	0.0036 (7)
C15	0.0536 (11)	0.0588 (11)	0.0218 (8)	0.0124 (9)	0.0124 (7)	0.0017 (7)
C16	0.0266 (7)	0.0273 (7)	0.0239 (7)	0.0032 (5)	0.0050 (5)	0.0015 (5)
C17	0.0262 (7)	0.0303 (7)	0.0244 (7)	0.0047 (5)	0.0048 (5)	0.0029 (5)
C18	0.0318 (7)	0.0344 (8)	0.0309 (8)	0.0044 (6)	0.0113 (6)	0.0032 (6)
C19	0.0307 (8)	0.0446 (9)	0.0430 (9)	0.0101 (7)	0.0136 (7)	0.0039 (7)
C20	0.0290 (8)	0.0465 (9)	0.0396 (9)	0.0112 (7)	0.0024 (7)	0.0063 (7)
C21	0.0321 (8)	0.0404 (8)	0.0248 (7)	0.0066 (6)	0.0016 (6)	0.0048 (6)
C22	0.0495 (10)	0.0573 (11)	0.0353 (9)	0.0183 (8)	0.0207 (8)	0.0068 (8)
C11	0.0551 (11)	0.0439 (10)	0.0288 (8)	0.0162 (8)	0.0098 (7)	0.0054 (7)
C12	0.0441 (10)	0.0616 (12)	0.0310 (9)	−0.0035 (8)	0.0141 (7)	−0.0066 (8)
C13	0.0647 (13)	0.0845 (16)	0.0359 (10)	0.0439 (12)	0.0064 (9)	−0.0132 (10)
C14	0.0554 (12)	0.0823 (15)	0.0344 (9)	0.0429 (11)	0.0035 (8)	−0.0074 (9)

### Geometric parameters (Å, º)

**Table:**

S1—O3	1.4370 (11)	C15—H15C	0.9800
S1—O2	1.4396 (11)	C16—C17	1.385 (2)
S1—C1	1.7356 (16)	C16—C21	1.393 (2)
S1—C16	1.7619 (16)	C17—C18	1.390 (2)
O1—C8	1.368 (2)	C17—H17	0.9500
O1—C7	1.3821 (18)	C18—C19	1.396 (2)
C1—C8	1.361 (2)	C18—C22	1.504 (2)
C1—C2	1.451 (2)	C19—C20	1.378 (2)
C2—C7	1.387 (2)	C19—H19	0.9500
C2—C3	1.398 (2)	C20—C21	1.380 (3)
C3—C4	1.394 (2)	C20—H20	0.9500
C3—H3	0.9500	C21—H21	0.9500
C4—C5	1.405 (2)	C22—H22A	0.9800
C4—C9	1.514 (2)	C22—H22B	0.9800
C5—C6	1.386 (2)	C22—H22C	0.9800
C5—H5	0.9500	C11—C12	1.504 (3)
C6—C7	1.373 (2)	C11—H11A	0.9900
C6—H6	0.9500	C11—H11B	0.9900
C8—C15	1.488 (2)	C12—C13	1.507 (3)
C9—C10	1.516 (2)	C12—H12A	0.9900
C9—C14	1.519 (2)	C12—H12B	0.9900
C9—H9	1.0000	C13—C14	1.533 (3)
C10—C11	1.526 (2)	C13—H13A	0.9900
C10—H10A	0.9900	C13—H13B	0.9900
C10—H10B	0.9900	C14—H14A	0.9900
C15—H15A	0.9800	C14—H14B	0.9900
C15—H15B	0.9800		
			
O3—S1—O2	119.03 (7)	C17—C16—C21	121.58 (15)
O3—S1—C1	108.30 (7)	C17—C16—S1	119.06 (11)
O2—S1—C1	107.58 (7)	C21—C16—S1	119.36 (12)
O3—S1—C16	108.82 (7)	C16—C17—C18	119.96 (13)
O2—S1—C16	107.73 (7)	C16—C17—H17	120.0
C1—S1—C16	104.43 (7)	C18—C17—H17	120.0
C8—O1—C7	106.82 (12)	C17—C18—C19	118.09 (15)
C8—C1—C2	107.39 (14)	C17—C18—C22	119.86 (14)
C8—C1—S1	126.89 (12)	C19—C18—C22	122.05 (15)
C2—C1—S1	125.71 (11)	C20—C19—C18	121.58 (16)
C7—C2—C3	119.35 (14)	C20—C19—H19	119.2
C7—C2—C1	104.61 (13)	C18—C19—H19	119.2
C3—C2—C1	136.03 (14)	C19—C20—C21	120.49 (15)
C4—C3—C2	118.79 (14)	C19—C20—H20	119.8
C4—C3—H3	120.6	C21—C20—H20	119.8
C2—C3—H3	120.6	C20—C21—C16	118.29 (15)
C3—C4—C5	119.38 (14)	C20—C21—H21	120.9
C3—C4—C9	121.55 (14)	C16—C21—H21	120.9
C5—C4—C9	119.06 (14)	C18—C22—H22A	109.5
C6—C5—C4	122.58 (15)	C18—C22—H22B	109.5
C6—C5—H5	118.7	H22A—C22—H22B	109.5
C4—C5—H5	118.7	C18—C22—H22C	109.5
C7—C6—C5	116.20 (15)	H22A—C22—H22C	109.5
C7—C6—H6	121.9	H22B—C22—H22C	109.5
C5—C6—H6	121.9	C12—C11—C10	111.21 (15)
C6—C7—O1	125.67 (15)	C12—C11—H11A	109.4
C6—C7—C2	123.70 (15)	C10—C11—H11A	109.4
O1—C7—C2	110.63 (14)	C12—C11—H11B	109.4
C1—C8—O1	110.55 (14)	C10—C11—H11B	109.4
C1—C8—C15	134.82 (16)	H11A—C11—H11B	108.0
O1—C8—C15	114.63 (14)	C11—C12—C13	111.00 (16)
C4—C9—C10	113.42 (13)	C11—C12—H12A	109.4
C4—C9—C14	112.13 (13)	C13—C12—H12A	109.4
C10—C9—C14	110.10 (15)	C11—C12—H12B	109.4
C4—C9—H9	106.9	C13—C12—H12B	109.4
C10—C9—H9	106.9	H12A—C12—H12B	108.0
C14—C9—H9	106.9	C12—C13—C14	111.88 (17)
C9—C10—C11	111.95 (14)	C12—C13—H13A	109.2
C9—C10—H10A	109.2	C14—C13—H13A	109.2
C11—C10—H10A	109.2	C12—C13—H13B	109.2
C9—C10—H10B	109.2	C14—C13—H13B	109.2
C11—C10—H10B	109.2	H13A—C13—H13B	107.9
H10A—C10—H10B	107.9	C9—C14—C13	111.20 (15)
C8—C15—H15A	109.5	C9—C14—H14A	109.4
C8—C15—H15B	109.5	C13—C14—H14A	109.4
H15A—C15—H15B	109.5	C9—C14—H14B	109.4
C8—C15—H15C	109.5	C13—C14—H14B	109.4
H15A—C15—H15C	109.5	H14A—C14—H14B	108.0
H15B—C15—H15C	109.5		
			
O3—S1—C1—C8	6.42 (17)	C7—O1—C8—C15	178.75 (14)
O2—S1—C1—C8	136.29 (14)	C3—C4—C9—C10	46.6 (2)
C16—S1—C1—C8	−109.42 (15)	C5—C4—C9—C10	−134.85 (16)
O3—S1—C1—C2	−174.83 (12)	C3—C4—C9—C14	−78.8 (2)
O2—S1—C1—C2	−44.96 (15)	C5—C4—C9—C14	99.67 (19)
C16—S1—C1—C2	69.33 (14)	C4—C9—C10—C11	177.73 (14)
C8—C1—C2—C7	−0.28 (16)	C14—C9—C10—C11	−55.7 (2)
S1—C1—C2—C7	−179.24 (12)	O3—S1—C16—C17	146.24 (12)
C8—C1—C2—C3	178.68 (17)	O2—S1—C16—C17	15.91 (14)
S1—C1—C2—C3	−0.3 (3)	C1—S1—C16—C17	−98.28 (13)
C7—C2—C3—C4	0.6 (2)	O3—S1—C16—C21	−34.28 (14)
C1—C2—C3—C4	−178.23 (16)	O2—S1—C16—C21	−164.62 (12)
C2—C3—C4—C5	−0.7 (2)	C1—S1—C16—C21	81.19 (13)
C2—C3—C4—C9	177.82 (13)	C21—C16—C17—C18	−0.8 (2)
C3—C4—C5—C6	0.2 (2)	S1—C16—C17—C18	178.65 (11)
C9—C4—C5—C6	−178.34 (15)	C16—C17—C18—C19	−0.3 (2)
C4—C5—C6—C7	0.4 (3)	C16—C17—C18—C22	179.37 (15)
C5—C6—C7—O1	178.77 (15)	C17—C18—C19—C20	1.1 (3)
C5—C6—C7—C2	−0.4 (2)	C22—C18—C19—C20	−178.58 (17)
C8—O1—C7—C6	−178.71 (16)	C18—C19—C20—C21	−0.8 (3)
C8—O1—C7—C2	0.58 (17)	C19—C20—C21—C16	−0.3 (3)
C3—C2—C7—C6	0.0 (2)	C17—C16—C21—C20	1.1 (2)
C1—C2—C7—C6	179.13 (15)	S1—C16—C21—C20	−178.35 (12)
C3—C2—C7—O1	−179.35 (13)	C9—C10—C11—C12	56.2 (2)
C1—C2—C7—O1	−0.18 (17)	C10—C11—C12—C13	−55.2 (2)
C2—C1—C8—O1	0.66 (17)	C11—C12—C13—C14	55.2 (2)
S1—C1—C8—O1	179.60 (11)	C4—C9—C14—C13	−177.83 (18)
C2—C1—C8—C15	−178.72 (18)	C10—C9—C14—C13	54.9 (2)
S1—C1—C8—C15	0.2 (3)	C12—C13—C14—C9	−55.4 (3)
C7—O1—C8—C1	−0.77 (17)		

### Hydrogen-bond geometry (Å, º)

Cg1 and Cg2 are the centroids of the C2–C7 benzene ring and the C1/C2/C7/O1/C8 furan ring, respectively

**Table:**

*D*—H···*A*	*D*—H	H···*A*	*D*···*A*	*D*—H···*A*
C17—H17···O2^i^	0.95	2.55	3.415 (2)	151
C13—H13*B*···*Cg*1^ii^	0.99	2.83	3.670 (2)	143
C19—H19···*Cg*2^iii^	0.95	2.85	3.705 (2)	150

Symmetry codes: (i) −*x*+1, −*y*, −*z*+1; (ii) −*x*+1, −*y*+1, −*z*+1; (iii) *x*−1, *y*, *z*.
